# Respiratory multiplex PCR and antimicrobial treatment modification in hospitalized patients with community-acquired pneumonia

**DOI:** 10.1017/ash.2026.10760

**Published:** 2026-07-22

**Authors:** Hülya Çaşkurlu, Halenur Vural Akbal, Hatice Kübra Uçar, Esra Koçoğlu, Özlem Aydın, Pınar Ergen, Yasemin Çağ

**Affiliations:** 1 Department of Infectious Diseases and Clinical Microbiology, Istanbul Medeniyet University Faculty of Medicine, Istanbul, Türkiye; 2 Department of Medical Microbiology, Istanbul Medeniyet University Faculty of Medicine, Istanbul, Türkiye

## Abstract

**Background:**

Community-acquired pneumonia (CAP) is a major cause of hospitalization, and pathogen identification is often limited by low diagnostic yield of conventional microbiological methods. Respiratory multiplex PCR (mPCR) enables rapid pathogen detection, but its role in antimicrobial treatment modification for inpatients with CAP remains unclear. This study evaluated the association between mPCR testing and antimicrobial modification in hospitalized patients with CAP.

**Methods:**

This retrospective, single center cohort study included hospitalized patients with CAP identified using ICD-10 codes between July 2023 and February 2025. Respiratory mPCR was performed within the first 24 hours of admission using combined nasopharyngeal/oropharyngeal (NP/OP) swabs. The primary outcome was antimicrobial treatment modification guided by mPCR results and documented by physicians. Secondary outcomes included antimicrobial de-escalation and hospital length of stay.

**Results:**

In this cohort of 145 patients, mPCR detected a pathogen in 56 patients (38.6%): viral pathogens in 30 (20.7%) and bacterial pathogens in 26 (17.9%). Overall antimicrobial treatment was modified in 54 patients (37.2%). Modification guided by mPCR results and documented by physicians occurred in 37 patients (25.5%). Among these 37 modifications, 35 (94.6%) were classified as antimicrobial de-escalation. Hospital length of stay did not differ significantly across mPCR results.

**Conclusions:**

In hospitalized patients with CAP, mPCR facilitates targeted antimicrobial treatment modification and drives high rates of de-escalation. These findings demonstrate the significant utility of mPCR in optimizing clinical decisions and enhancing antimicrobial stewardship.

## Introduction

CAP is one of the most common acute lower respiratory tract infections and remains associated with substantial morbidity and mortality.^1–3^ The diagnosis is usually based on compatible clinical findings together with radiological evidence of a new pulmonary infiltrate on chest radiography, computed tomography, or lung ultrasound.^4,5^ However, identifying the causative pathogen remains difficult in many hospitalized patients with CAP. In routine practice, this uncertainty often leads to empirical antimicrobial treatment and may limit opportunities for early antimicrobial adjustment.^6,7^

Several microbiological methods are used to identify the etiology of CAP, including blood and respiratory cultures, urinary antigen testing, serology, and nucleic acid amplification tests such as PCR.^3^ Although culture-based results may support antimicrobial adjustment, conventional cultures are time-consuming, frequently have low positivity rates, and depend on specimen quality and prior antimicrobial exposure.^8,9^ In addition, inadequate sputum production is common in hospitalized patients, which further limits the usefulness of conventional respiratory cultures.

Respiratory mPCR assays provide faster detection of multiple viral and bacterial pathogens and may support antimicrobial stewardship by allowing earlier treatment adjustment.^10,11^ Recent guidelines for CAP emphasize the potential role of molecular testing in improving diagnostic accuracy and supporting de-escalation or discontinuation of antimicrobial therapy when clinically appropriate.^12^ However, these recommendations and much of the supporting evidence mainly concern lower respiratory tract specimens, which may better reflect the site of infection.

In daily practice, combined NP/OP swabs are easier to obtain early after admission and may allow more timely mPCR testing. Nevertheless, the clinical data regarding the validity of upper respiratory tract swabs in CAP, especially their diagnostic sensitivity, are far more limited compared to lower respiratory tract sampling. Therefore, their interpretation requires caution, as detecting a pathogen in an upper respiratory tract sample may not always indicate a true lower respiratory tract infection. The clinical value of mPCR also depends on how results are interpreted within the broader clinical context. Delays in appropriate treatment, overdiagnosis of pneumonia, and misinterpretation of microbiological findings may all contribute to unnecessary or suboptimal antibiotic use.^13,14^ In hospitalized patients, treatment decisions and clinical outcomes may also be influenced by age, comorbidity burden, disease severity, oxygen requirement, and noninfectious conditions that can mimic pneumonia.^15^ Therefore, the role of respiratory tract mPCR in guiding antimicrobial treatment decisions among hospitalized patients with CAP remains unclear.

This study evaluated the association between early respiratory mPCR testing using combined NP/OP swabs and physician-documented antimicrobial treatment modification among hospitalized adults with CAP. Secondary objectives were to assess antimicrobial de-escalation among modifications guided by mPCR results, hospital length of stay, and exploratory associations between mPCR result categories and radiological patterns.

## Materials and methods

### Study design and population

This retrospective cohort study was conducted at the Infectious Diseases Clinic of Istanbul Medeniyet University, Göztepe Prof. Dr. Süleyman Yalçın City Hospital. Patients aged ≥18 years who were hospitalized with CAP between July 2023 and February 2025 were screened for eligibility. Eligible patients were identified from electronic hospital records using International Classification of Diseases, Tenth Revision (ICD-10) codes. CAP was defined as the presence of a new pulmonary infiltrate on chest imaging consistent with pneumonia, together with compatible clinical findings of lower respiratory tract infection at presentation or within the first 48 hours of admission.^10^ These included cough, sputum production, dyspnea, tachypnea, fever or hypothermia, abnormal lung auscultation, leukocytosis or leukopenia.

**Figure 1.**
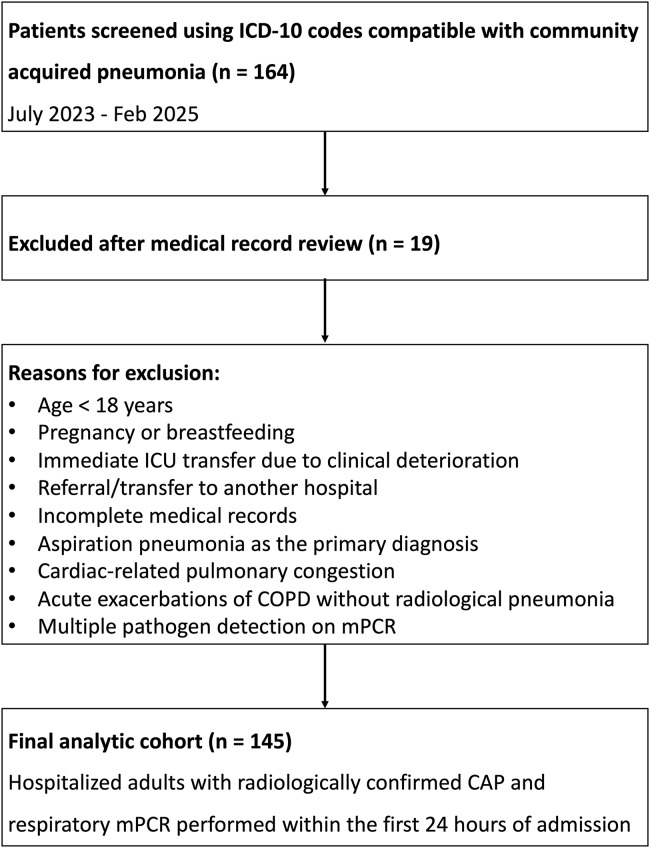
Patient selection flowchart for the final analytic cohort.

Patients were included if they underwent mPCR testing within 24 hours of admission. Exclusion criteria included pregnancy or breastfeeding, immediate intensive care unit transfer due to acute clinical deterioration, interfacility transfer, incomplete medical records, an alternative primary diagnosis (eg, cardiogenic pulmonary edema), aspiration pneumonia, acute exacerbations of chronic obstructive pulmonary disease or asthma, and multiple pathogen detection on the mPCR panel. Aspiration pneumonia was considered when physician documentation identified aspiration as the main cause, supported by a history aspiration, swallowing dysfunction, impaired consciousness, or radiological findings predominantly compatible with dependent infiltrates. After applying these criteria, the final analytic cohort consisted of 145 patients (Figure 1).

The primary outcome was antimicrobial treatment modification guided by mPCR results and documented by physicians. Secondary outcomes included antimicrobial de-escalation and hospital length of stay. The association between mPCR result categories and the main radiological patterns was evaluated as an exploratory analysis.

### Data collection

Demographic, clinical, laboratory, radiological, microbiological, and treatment related data were retrospectively extracted from electronic medical records. Demographic and clinical variables included age, sex, chronic comorbidities, documented influenza and pneumococcal vaccination status, admission vital signs, and respiratory support requirement. Laboratory variables included white blood cell count, neutrophil to white blood cell ratio, absolute lymphocyte count, and C-reactive protein level at admission. Prior antibiotic exposure was recorded when systemic antibiotic use within 10 days before admission was noted. Vaccination status was based on documented influenza vaccination within the previous year and pneumococcal vaccination within the previous 10 years.

Radiological findings were extracted from chest radiography or computed tomography reports and categorized according to the main pattern as ground-glass opacities, lobar pneumonia, or diffuse parenchymal involvement. Microbiological data included mPCR results, detected pathogens, blood culture results, and sputum culture results. Respiratory mPCR results were categorized as negative, viral pathogen detected, or bacterial pathogen detected.

Antimicrobial treatment modification was defined as any change in the initial empirical regimen, including de-escalation, escalation, discontinuation, or targeted antimicrobial change. A modification was considered guided by mPCR when the treating physician explicitly documented the mPCR result as the reason for the change. Modifications guided by clinical findings were defined as changes attributed to clinical deterioration, clinical improvement, or allergic reaction. Antimicrobial de-escalation was defined as narrowing of the antimicrobial spectrum, discontinuation of at least one antibacterial or antiviral agent, or cessation of empirical therapy when clinically appropriate.

### Respiratory mPCR testing

Combined NP/OP swab specimens were collected within the first 24 hours of admission and transported to the laboratory in viral transport medium according to CDC Standard Operating Procedure (SOP#: DSR-052-05). Testing was performed using a commercially available BioSpeedy® respiratory pathogen mPCR panel according to the manufacturer’s instructions. Nucleic acid extraction from these samples was performed using a rapid nucleic acid extraction kit (BioSpeedy®, Cat. No: ZFNAE01) on the Zybio EXM3000 automated extraction system. Reverse transcription and real-time polymerase chain reaction analyses were carried out on the CFX96 Real-Time PCR Detection System (Bio-Rad, USA) in accordance with the manufacturer’s recommended protocol.

Test interpretation and analysis were performed based on the manufacturer’s predefined evaluation criteria. The mPCR panel was designed to detect the following respiratory pathogens: SARS-CoV-2; human coronaviruses 229E, OC43, NL63, and HKU1; human parainfluenza viruses types 1–4; human metapneumovirus; human enterovirus; human adenovirus; influenza A and B viruses, including influenza A H1, H3, and H1N1-2009 subtypes; human bocavirus; human rhinovirus; respiratory syncytial virus A/B; and the bacterial pathogens *Legionella pneumophila, Streptococcus pyogenes, Mycoplasma pneumoniae, Haemophilus influenzae, Bordetella pertussis,* and *Streptococcus pneumoniae.*

### Statistical analysis

Statistical analyses were performed using Jamovi software, version 2.7.^16^ Continuous variables were assessed for normality using the Shapiro–Wilk test and are presented as mean ± SD. Because variables did not meet normality assumptions, nonparametric tests were used: the Mann–Whitney U test for comparisons between two groups and the Kruskal–Wallis test for comparisons across more than two groups. These tests were applied to analyze hospital length of stay across mPCR categories and comorbidity status. Categorical variables were summarized as counts and percentages, and compared using the χ^2^ test. This included evaluating the associations between mPCR result categories, antimicrobial modifications guided by mPCR, and radiological patterns, with the radiological analysis considered exploratory. All tests were two tailed, and *P* < .05 was considered statistically significant.

## Results

### Patient characteristics

The final analysis included 145 hospitalized adults with CAP. The mean age was 71.9 ± 17.1 years, and 76 patients (52.4%) were male. At least one chronic comorbidity was present in 132 patients (91.0%), with hypertension, diabetes mellitus, and coronary artery disease being the most frequent. Prior antibiotic exposure within 10 days of admission was documented in 72 patients (49.7%). At presentation, the mean body temperature was 36.7 ± 0.5°C, and most patients (66.2%) were breathing room air, whereas 34 (23.4%) required a nasal cannula and 15 (10.4%) required an oxygen mask. Baseline demographic, clinical, laboratory, preadmission antibiotic exposure, and vaccination data are summarized in Table 1. Data are presented as n (%) or mean ± standard deviation (SD). Percentages were calculated using the overall cohort as the denominator.

**Table 1.** Demographic and clinical characteristics of the study cohort Table 1 long description.

Characteristic	Overall cohort (n = 145)
**Demographic characteristics**	
Age, years	71.9 ± 17.1
Male sex, n (%)	76 (52.4)
**Comorbidity profile**	
≥1 chronic comorbidity, n (%)	132 (91.0)
Hypertension, n (%)	90 (62.1)
Diabetes mellitus, n (%)	48 (33.1)
Coronary artery disease, n (%)	48 (33.1)
Dementia/Alzheimer disease, n (%)	28 (19.3)
Immunosuppression, n (%)	24 (16.6)
Previous cerebrovascular disease, n (%)	18 (12.4)
Atrial fibrillation, n (%)	17 (11.7)
Chronic kidney disease, n (%)	13 (9.0)
Epilepsy, n (%)	9 (6.2)
**Preadmission and vaccination status**	
Prior antibiotic exposure, n (%)	72 (49.7)
Documented influenza vaccination, n (%)	7 (4.8)
Documented pneumococcal vaccination, n (%)	45 (31.0)
**Admission vital signs**	
Body temperature, °C	36.7 ± 0.5
Mean arterial pressure, mmHg	92.9 ± 14.1
Heart rate, beats/min	91.0 ± 16.2
Respiratory rate, breaths/min	24.3 ± 5.2
**Respiratory support at admission**	
Room air, n (%)	96 (66.2)
Nasal cannula, n (%)	34 (23.4)
Oxygen mask, n (%)	15 (10.4)
**Laboratory parameters at admission**	
White blood cell count (WBC), cells/mm^3^	12,643 ± 6,456
Neutrophil/WBC ratio	0.83 ± 0.23
Lymphocyte count, cells/mm^3^	1,192 ± 727
C-reactive protein, mg/L	136.4 ± 113.1
**Clinical outcome**	
Length of stay, days	6.4 ± 2.3

### Microbiological and radiological findings

The mPCR detected a pathogen in 56 patients (38.6%): viral agents in 30 (20.7%) and bacterial agents in 26 (17.9%). Influenza virus and SARS CoV 2 were the most common viral pathogens, whereas *Streptococcus pneumoniae* and *Haemophilus influenzae* predominated among bacterial species. Conventional cultures provided limited additional information; blood culture growth consisted entirely of skin commensals considered clinically insignificant, and sputum cultures were available for 23 patients (15.9%), most commonly showed upper respiratory tract flora. Radiological evaluation showed ground glass opacities in 72 patients (49.7%), lobar pneumonia in 48 (33.1%), and diffuse parenchymal involvement in 25 (17.2%). These findings are presented in Table 2.

**Table 2.** mPCR findings, conventional culture, and radiological pattern results Table 2 long description.

Finding	Overall cohort (n = 145)
**mPCR result category**	
Negative	89 (61.4)
Any pathogen detected	56 (38.6)
Viral pathogen detected	30 (20.7)
Bacterial pathogen detected	26 (17.9)
**Viral pathogens**	
Influenza virus	16 (11.0)
SARS CoV-2	8 (5.5)
Parainfluenza virus	2 (1.4)
Coronavirus	2 (1.4)
Respiratory syncytial virus	1 (0.7)
Rhinovirus	1 (0.7)
**Bacterial pathogens**	
*Streptococcus pneumoniae*	14 (9.7)
*Haemophilus influenzae*	11 (7.6)
*Mycoplasma pneumoniae*	1 (0.7)
**Conventional culture findings**	
**Blood culture with no growth**	93 (64.1)
**Blood culture growth of skin commensals**	52 (35.9)
*Staphylococcus hominis*	33 (22.8)
*Staphylococcus epidermidis*	12 (8.3)
*Staphylococcus capitis*	5 (3.4)
*Staphylococcus haemolyticus*	2 (1.4)
**Sputum culture findings**	23 (15.9)
Upper respiratory tract flora	15 (10.3)
*Candida albicans*	6 (4.1)
*Streptococcus pneumoniae*	1 (0.7)
*Saprochaete capitata*	1 (0.7)
**Radiological pattern**	
Ground glass opacities	72 (49.7)
Lobar pneumonia	48 (33.1)
Diffuse parenchymal involvement	25 (17.2)

**Figure 2.**
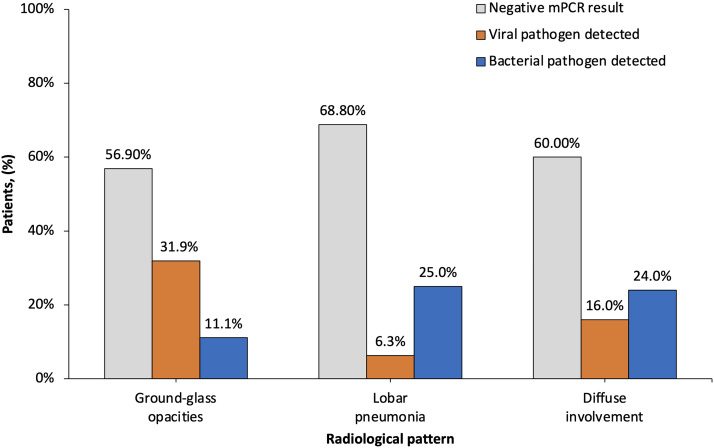
Distribution of mPCR results according to radiological pattern.

In an exploratory analysis, the mPCR result category was significantly associated with the radiological pattern (*P* = .028, chi square test). Viral detection was more frequent among patients with ground glass opacities, whereas bacterial pathogens were more common in those with lobar pneumonia or diffuse parenchymal involvement (Figure 2).

### Antimicrobial treatment modification

Overall, antimicrobial treatment was modified in 54 patients (37.2%). Of these, 37 modifications (25.5% of the overall cohort) were guided by mPCR and documented by physicians, whereas the remaining 17 (11.7%) were guided by clinical findings. This primary outcome differed significantly across mPCR results (*P* < .001, chi square test); modification occurred in 8 of 89 patients (9.0%) with negative results, 11 of 30 (36.7%) with viral pathogens, and 18 of 26 (69.2%) with bacterial pathogens. These outcomes are summarized in Table 3.

**Table 3.** Antimicrobial treatment modification according to mPCR results Table 3 long description.

mPCR result category	Total patients (n)	Any modification, n (%)	Modification guided by mPCR, n (%)	Modification guided by clinical findings, n (%)
Negative result	89	19 (21.3)	8 (9.0)	11 (12.4)
Viral pathogen detected	30	15 (50.0)	11 (36.7)	4 (13.3)
Bacterial pathogen detected	26	20 (76.9)	18 (69.2)	2 (7.7)
Overall cohort	145	54 (37.2)	37 (25.5)	17 (11.7)

Among the 37 modifications guided by mPCR, 35 (94.6%) were classified as antimicrobial de-escalation, representing 24.1% of the overall cohort. De-escalation included narrowing the antimicrobial spectrum or discontinuing at least one antibacterial or antiviral agent when clinically appropriate. These findings indicate that positive mPCR results were associated with documented antimicrobial modifications, primarily through de-escalation. Data are presented as n (%), with percentages calculated using the row denominator. Modifications guided by clinical findings included clinical deterioration (n = 10), clinical improvement (n = 3), and allergic reaction (n = 4).

### Hospital length of stay

The mean hospital length of stay was 6.4 ± 2.3 days. Hospital length of stay did not differ significantly across mPCR results (*P* = .177, Kruskal Wallis test). Patients with at least one chronic comorbidity had a longer hospital stay than those without comorbidities (6.5 ± 2.2 vs 5.3 ± 1.8 d; *P* = .049, Mann Whitney *U* test). This finding should be interpreted conservatively because only 13 patients had no chronic comorbidity.

## Discussion

This study showed that mPCR testing was associated with antimicrobial treatment modification in hospitalized patients with CAP. A pathogen was detected in 38.6% of patients, and mPCR-guided, physician-documented antimicrobial modification occurred in 25.5% of the overall cohort. The frequency of antimicrobial modifications increased significantly across mPCR categories, rising from 9.0% in negative cases to 36.7% in viral and 69.2% in bacterial pathogen detection groups. Among modifications guided by mPCR results, 94.6% were classified as antimicrobial de-escalation.

The pathogen distribution observed in this cohort reflects the heterogeneous etiology of CAP and the diagnostic uncertainty frequently encountered in routine practice. Viral pathogens were detected slightly more often than bacterial pathogens, with influenza virus and SARS-CoV-2 being the most common viral agents. Among bacterial pathogens, *Streptococcus pneumoniae* and *Haemophilus influenzae* predominated, consistent with their established role as common bacterial causes of CAP. However, more than half of the cohort had negative mPCR results. This finding may partly be explained by prior antibiotic exposure, as nearly half of the mPCR-negative patients had documented antibiotic use within 10 days before admission. Conventional sputum culture provided limited additional information, as samples were obtained from only a small proportion of patients and mostly yielded upper respiratory tract flora. This finding reflects the practical difficulty of obtaining adequate sputum specimens in hospitalized patients with CAP.

The most clinically relevant finding was the association between mPCR results and antimicrobial modification. Pathogen detected mPCR results were more often associated with antimicrobial treatment modification. Although previous studies suggest that molecular respiratory testing supports antimicrobial stewardship by facilitating treatment optimization, its impact varies across clinical settings.^17–20^ In this cohort, most modifications guided by mPCR results were classified as de-escalation. In contrast, negative mPCR results less frequently led to treatment adjustments, suggesting that a negative panel may not provide clinicians with sufficient confidence to discontinue empirical therapy in hospitalized patients with CAP.

The interpretation of mPCR results depends on the respiratory specimen type. In this study, mPCR was performed using combined NP/OP swabs collected within 24 hours of admission. Although current guidelines emphasize lower respiratory tract specimens because they more directly represent the site of infection,^12^ NP/OP swabs remain highly practical in routine clinical practice due to their rapid, noninvasive nature, especially when adequate sputum is unavailable. However, detection of a bacterial pathogen in an upper respiratory tract sample may reflect colonization. Similarly, a negative NP/OP result does not exclude lower respiratory tract infection. Therefore, mPCR findings should be interpreted as supportive microbiological data and should be evaluated together with clinical, laboratory, and radiological findings.

The exploratory radiological analysis showed an association between mPCR result category and radiological pattern. Viral pathogen detection was more frequent among patients with ground-glass opacities, whereas bacterial pathogen detection was more common among patients with lobar pneumonia or diffuse parenchymal involvement. These findings are broadly consistent with previous radiological descriptions of viral and bacterial pneumonia.^21,22^ However, this analysis was retrospective, included a limited sample size, and was not designed to develop or validate a predictive model. Therefore, radiological pattern should not be used alone to predict mPCR results or guide antimicrobial decisions.

Hospital length of stay did not differ significantly across mPCR result categories. Because all included patients underwent mPCR testing, this study cannot determine whether mPCR testing changed hospital length of stay compared with an untested comparator group. Rather, it indicates that length of stay was similar among patients with negative, viral, and bacterial mPCR results within the tested cohort. Patients with at least one chronic comorbidity had a longer hospital stay than those without chronic comorbidity; however, this finding is limited by the small number of patients without chronic comorbidity. Length of stay in CAP is influenced by multiple factors beyond microbiological diagnosis, including comorbidity burden, oxygen requirement, clinical stability, and discharge planning.

This study has several limitations. First, its retrospective, single-center design limits causal inference and generalizability. Second, there was no comparator group of patients managed without mPCR testing; therefore, the direct effect of mPCR on antimicrobial use or length of stay could not be measured. Moreover, mPCR testing was performed using combined NP/OP swabs rather than lower respiratory tract specimens. In addition, patients with multiple pathogen detection were excluded, which may limit generalizability to CAP cases with co-detection. Despite these limitations, this study provides clinically relevant data on how mPCR results were incorporated into antimicrobial treatment decisions in hospitalized patients with CAP.

Overall, these findings suggest that mPCR testing may support antimicrobial treatment modification in hospitalized patients with CAP, mainly by facilitating de-escalation when a pathogen is detected. However, its clinical value depends on specimen type, result interpretation, and the broader clinical context. Respiratory mPCR results should therefore be interpreted alongside clinical and radiographic findings rather than used alone. Finally, future prospective studies with control groups are needed to better clarify the true impact of mPCR testing on antimicrobial utilization and clinical outcomes.

## Conclusion

In conclusion, mPCR testing was associated with antimicrobial modifications in hospitalized patients with CAP, predominantly through treatment de-escalation following pathogen detection. These findings support the role of mPCR as an antimicrobial stewardship tool in routine clinical practice. However, because testing was performed on upper respiratory tract samples, the results should be interpreted together with clinical and radiological findings. Future prospective studies with control groups are needed to determine the impact of mPCR on antimicrobial utilization and clinical outcomes.

## Data Availability

The datasets analyzed during the current study are available from the corresponding author on reasonable request.

## Table 1. Long description

A table summarizing demographic and clinical characteristics of a study cohort. The table has 14 rows and 2 columns. The columns are labeled ‘Characteristic’ and ‘Overall cohort (n = 145)’. The rows are grouped into categories: Demographic characteristics, Comorbidity profile, Preadmission and vaccination status, Admission vital signs, Respiratory support at admission, Laboratory parameters at admission, and Clinical outcome. Each row lists a specific characteristic and its corresponding value or percentage. For example, the mean age is 71.9 ± 17.1 years, and 76 patients (52.4%) are male. The table includes various health conditions, vaccination statuses, vital signs, respiratory support details, laboratory parameters, and clinical outcomes.

Navigate back to Table 1..

## Table 2. Long description

A table summarizing mPCR findings, conventional culture, and radiological patterns in a cohort of 145 patients. The table has 27 rows and 2 columns. The columns are labeled ‘Finding’ and ‘Overall cohort (n = 145)’. The table is divided into several sections: mPCR result category, Viral pathogens, Bacterial pathogens, Conventional culture findings, Sputum culture findings, and Radiological pattern. Each section lists specific findings and their corresponding percentages. For example, under mPCR result category, 61.4 percent of patients had negative results, 38.6 percent had any pathogen detected, 20.7 percent had a viral pathogen detected, and 17.9 percent had a bacterial pathogen detected. The Viral pathogens section lists specific viruses and their detection percentages, such as Influenza virus at 11.0 percent and SARS CoV-2 at 5.5 percent. The Bacterial pathogens section lists specific bacteria and their detection percentages, such as Streptococcus pneumoniae at 9.7 percent and Haemophilus influenzae at 7.6 percent. The Conventional culture findings section includes blood culture results, with 64.1 percent showing no growth and 35.9 percent showing growth of skin commensals. The Sputum culture findings section lists results such as upper respiratory tract flora at 10.3 percent and Candida albicans at 4.1 percent. The Radiological pattern section includes findings like ground glass opacities at 49.7 percent, lobar pneumonia at 33.1 percent, and diffuse parenchymal involvement at 17.2 percent.

Navigate back to Table 2..

## Table 3. Long description

A table comparing antimicrobial treatment modifications based on mPCR results. The table has four rows and four columns. The columns are labeled Total patients (n), Any modification, n (%), Modification guided by mPCR, n (%), and Modification guided by clinical findings, n (%). The rows are labeled Negative result, Viral pathogen detected, Bacterial pathogen detected, and Overall cohort. Row 1: Total patients, 89; Any modification, 19 (21.3%); Modification guided by mPCR, 8 (9.0%); Modification guided by clinical findings, 11 (12.4%). Row 2: Total patients, 30; Any modification, 15 (50.0%); Modification guided by mPCR, 11 (36.7%); Modification guided by clinical findings, 4 (13.3%). Row 3: Total patients, 26; Any modification, 20 (76.9%); Modification guided by mPCR, 18 (69.2%); Modification guided by clinical findings, 2 (7.7%). Row 4: Total patients, 145; Any modification, 54 (37.2%); Modification guided by mPCR, 37 (25.5%); Modification guided by clinical findings, 17 (11.7%).

Navigate back to Table 3..

## References

[1] World Health Organization: WHO, Pneumonia in children. 2022. https://www.who.int/news-room/fact-sheets/detail/pneumonia.

[2] Torres A , Cilloniz C , Niederman MS , et al. Pneumonia. Nat Rev Dis Primers 2021;7:25. 10.1038/s41572-021-00259-0. 33833230

[3] Aliberti S , Cruz CSD , Amati F , Sotgiu G , Restrepo MI . Community-acquired pneumonia. Lancet 2021;398:906–919. 10.1016/s0140-6736(21)00630-9. 34481570

[4] Staub LJ , Biscaro RRM , Kaszubowski E , Maurici R. Lung ultrasound for the emergency diagnosis of pneumonia, acute heart failure, and exacerbations of chronic obstructive pulmonary disease/asthma in adults: a systematic review and meta-analysis. J Emerg Med 2018;56:53–69. 10.1016/j.jemermed.2018.09.009. 30314929

[5] Moore M , Stuart B , Little P , et al. Predictors of pneumonia in lower respiratory tract infections: 3C prospective cough complication cohort study. Eur Respir J 2017;50:1700434. 10.1183/13993003.00434-2017. 29167296 PMC5724402

[6] Carugati M , Aliberti S , Reyes LF , et al. Microbiological testing of adults hospitalised with community-acquired pneumonia: an international study. ERJ Open Research 2018;4:00096–02018. 10.1183/23120541.00096-2018. 30474036 PMC6174282

[7] Vaughn VM , Flanders SA , Snyder A , et al. Excess antibiotic treatment duration and adverse events in patients hospitalized with pneumonia. Ann Intern Med 2019;171:153–163. 10.7326/m18-3640. 31284301 PMC13258130

[8] Metlay JP , Waterer GW , Long AC , et al. Diagnosis and treatment of adults with community-acquired pneumonia. An official clinical practice guideline of the American Thoracic Society and Infectious Diseases Society of America. Am J Respir Crit Care Med 2019;200:e45–e67. 10.1164/rccm.201908-1581st. 31573350 PMC6812437

[9] Jain S , Self WH , Wunderink RG , et al. Community-acquired pneumonia requiring hospitalization among U.S. Adults. N Engl J Med 2015;373:415–427. 10.1056/nejmoa1500245. 26172429 PMC4728150

[10] Hanson KE , Azar MM , Banerjee R , et al. Molecular testing for acute respiratory tract infections: clinical and diagnostic recommendations from the IDSA’s Diagnostics Committee. Clin Infect Dis 2020;71:2744–2751. 10.1093/cid/ciaa508. 32369578 PMC7454374

[11] Enne Aydin , Baldan A , Owen R , et al. Multicentre evaluation of two multiplex PCR platforms for the rapid microbiological investigation of nosocomial pneumonia in UK ICUs: the INHALE WP1 study. Thorax 2022;77:1220–1228. 10.1136/thoraxjnl-2021-216990. 35027473

[12] Martin-Loeches I , Torres A , Nagavci B , et al. ERS/ESICM/ESCMID/ALAT guidelines for the management of severe community-acquired pneumonia. Intensive Care Med 2023;49:615–632. 10.1007/s00134-023-07033-8. 37012484 PMC10069946

[13] Soper NS , Albin OR. Healthcare providers consistently overestimate the diagnostic probability of ventilator-associated pneumonia. Infect Control Hosp Epidemiol 2023;44:1927–1931. 10.1017/ice.2023.62. 37350254 PMC10755149

[14] Gupta AB , Flanders SA , Petty LA , et al. Inappropriate diagnosis of pneumonia among hospitalized adults. JAMA Intern Med 2024;184:548. 10.1001/jamainternmed.2024.0077. 38526476 PMC10964165

[15] Faverio P , Aliberti S , Bellelli G , et al. The management of community-acquired pneumonia in the elderly. Eur J Intern Med 2014;25:312–319. doi: 10.1016/j.ejim.2013.12.001. Epub 2013 Dec 17. PMID: 24360244; PMCID: PMC4102338. 24360244 PMC4102338

[16] The jamovi project. jamovi. (Version 2.7) [Computer Software]. 2025. https://www.jamovi.org.

[17] Abelenda-Alonso G , Calatayud L , Rombauts A , et al. Multiplex real-time PCR in non-invasive respiratory samples to reduce antibiotic use in community-acquired pneumonia: a randomised trial. Nat Commun 2024;15:7098. 10.1038/s41467-024-51547-8. 39154071 PMC11330507

[18] Miller MM , Van Schooneveld TC , Stohs EJ , et al. Implementation of a rapid multiplex polymerase chain reaction pneumonia panel and subsequent antibiotic de-escalation. Open Forum Infect Dis 2023;10:ofad382. 10.1093/ofid/ofad382. 37564742 PMC10411041

[19] Contier J , Platon L , Benchabane N , et al. Diagnostic performance of Pneumonia multiplex PCR in critically ill immunocompromised patients. Crit Care 2025;29:310. 10.1186/s13054-025-05528-y. 40676679 PMC12272964

[20] Aissaoui Y , Derkaoui A , Hachimi A , et al. Diagnostic performance and impact on antimicrobial treatment of a multiplex polymerase chain reaction in critically ill patients with pneumonia: a multicenter observational study (The MORICUP-PCR study (The MORICUP-PCR Study: Morocco ICU Pneumonia-PCR study). Crit Care Explor 2025;7:e1220. 10.1097/cce.0000000000001220. 39937572 PMC11826045

[21] Kim JE , Kim UJ , Kim HK , et al. Predictors of viral Pneumonia in patients with community-acquired Pneumonia. PLoS One 2014;9:e114710. 10.1371/journal.pone.0114710. 25531901 PMC4273967

[22] Nicolini A , Ferrera L , Rao F , Senarega R , Ferrari-Bravo M. Chest radiological findings of influenza A H1N1 pneumonia. Revista Portuguesa De Pneumologia 2012;18:120–127. 10.1016/j.rppnen.2011.12.006. 22483844

